# CHD8 safeguards early neuroectoderm differentiation in human ESCs and protects from apoptosis during neurogenesis

**DOI:** 10.1038/s41419-021-04292-5

**Published:** 2021-10-22

**Authors:** Song Ding, Xianchun Lan, Yajing Meng, Chenchao Yan, Mao Li, Xiang Li, Jian Chen, Wei Jiang

**Affiliations:** 1 Department of Biological Repositories, Frontier Science Center for Immunology and Metabolism, Medical Research Institute, Zhongnan Hospital of Wuhan University, Wuhan University, 430071 Wuhan, China; 2 Department of Neurosurgery, China Brain Research Center, Medical Research Institute, Zhongnan Hospital of Wuhan University, 430071 Wuhan, China; 3 Chinese Institute for Brain Research (Beijing), Research Unit of Medical Neurobiology, Chinese Academy of Medical Sciences, 102206 Beijing, China; 4 Institute of Functional Nano & Soft Materials (FUNSOM), Soochow University, 215123 Suzhou, China; 5 Human Genetics Resource Preservation Center of Wuhan University, 430071 Wuhan, China; 6 Hubei Provincial Key Laboratory of Developmentally Originated Disease, 430071 Wuhan, China

**Keywords:** Differentiation, Autism spectrum disorders, Embryonic stem cells

## Abstract

The chromatin remodeler *CHD8*, which belongs to the ATP-dependent chromatin remodelers CHD family, is one of the most high-risk mutated genes in autism spectrum disorders. However, the role of CHD8 in neural differentiation and the mechanism of CHD8 in autism remains unclear, despite there are a few studies based on the CHD8 haploinsufficient models. Here, we generate the CHD8 knockout human ESCs by CRISPR/Cas9 technology and characterize the effect of loss-of-function of CHD8 on pluripotency maintenance and lineage determination by utilizing efficient directed differentiation protocols. The results show loss-of-function of CHD8 does not affect human ESC maintenance although having slight effect on proliferation and cell cycle. Interestingly, CHD8 depletion results in defective neuroectoderm differentiation, along with severe cell death in neural progenitor stage. Transcriptome analysis also indicates CHD8 does not alter the expression of pluripotent genes in ESC stage, but in neural progenitor cells depletion of CHD8 induces the abnormal expression of the apoptosis genes and suppresses neuroectoderm-related genes. These results provide the evidence that CHD8 plays an essential role in the pluripotency exit and neuroectoderm differentiation as well as the regulation of apoptosis during neurogenesis.

## Introduction

Human embryonic stem cells (ESCs), derived from the inner cell mass of blastocysts, possess indefinite proliferative capacity and can differentiate into all three germ layers cell type. As such, they can serve as a model for development and disease studies, and as the ideal resource for regenerative medicine and drug screening [1]. Whether ESCs maintain the self-renewal or go to specific lineage differentiation largely depends on epigenetic regulation, including DNA and histone modifications as well as chromatin remodeling [2–4]. The key components of chromatin remodeling complexes are the remodeling enzymes, which contain an ATPase domain and can use the energy by hydrolyzing ATP to alter chromatin structure [5]. These ATP-dependent chromatin remodelers can be grouped into four major families by the flanking domains of the conserved ATPase domain: CHD, SWI/SNF, ISWI and INO80 family [6].

*CHD8* (Chromodomain-Helicase-DNA-binding protein 8), a member of CHD family that is characterized by a SNF2-like ATPase domain and two chromatin organization modifier domains [7], is identified as one of the most high-risk mutated genes in autism spectrum disorders (ASD) [8–12]. CHD8 was first described as a negative regulator of the WNT/β-catenin signaling by competitively binding the β-catenin in mouse cell line [13]. CHD8 played vital functions in embryonic and brain development, indicated by the report that the CHD8 knockout mice were embryonic lethal and arrested at gastrulation with massive apoptosis at embryonic day 7.5 [14]. However, another study based on knockdown of *CHD8* in cortical progenitors at the E13 mouse embryo by in utero electroporation showed depletion of CHD8 reduced the cell proliferation and WNT signaling genes of neural progenitors with downregulated cell cycle [15]. In addition, during neuronal differentiation from mouse neural stem cells, CHD8 was reported to be recruited by SMAD3 together with histone demethylase KDM6B to create appropriate chromatin environment to activate posterior gene transcription [16]. In ASD patients with *CHD8* de novo mutation, neurodevelopmental disorders and macrocephaly were observed, indicating CHD8 is associated with the brain development in human [17, 18]. Very interestingly, the CHD8 heterozygous knockout mice showed ASD-like phenotypes including macrocephaly and abnormal craniofacial, further supporting that CHD8 is an important player for ASD pathogenesis [19]. Cotney and colleagues [20] reported that CHD8 could directly bind to the promoter region of ASD-associated genes, which are enriched with euchromatin markers H3K4me3 and H3K27ac as well, suggesting CHD8 may act as a transcriptional activator. Besides of rodent model, scientists have also utilized human ESCs/induced pluripotent stem cells (iPSCs) and the derived neural lineage model as well as neural progenitor cell (NPC) lines to study the role of CHD8 in neural development. For example, by knockdown, Sugathan and colleagues [21] found that reduction of *CHD8* in human NPC line GM88330-8 mainly altered neurodevelopmental pathways and expression of ASD-related genes without affecting NPC morphology. In addition, human iPSC lines with *CHD8* de novo mutation reported in ASD patients or heterozygous CHD8 were generated [22–24]. The NPCs and differentiated neurons derived from these CHD8-defective iPSCs exhibited the altered expression pattern of genes associated with neurodevelopment and ASD. More interestingly, the CHD8 defect affected more genes in neurons, indicating CHD8 had continuous effect on neural development. By analyzing the cerebral organoids generated from the heterogenous iPSCs, CHD8 was found to not only regulate the genes related to ASD, but also affect the genes associated with the head volume, which also referred to ASD phonotype [22–24]. These studies together indicated CHD8 was functional in neural development. However, a conventional knockout model is emergingly required to dissect the cellular function and molecular mechanisms of CHD8 during the neurogenesis and ASD pathogenesis in detail.

Here, we use the CRISPR/Cas9 gene-editing technology to generate CHD8 knockout human ESC lines. The generated knockout ESC lines can be stably maintained for many passages. With the knockout ESCs, we dissect the functional role of CHD8 in human ESCs and neuroectoderm differentiation.

## Results

### Generation and characterization of CHD8 knockout human ESCs

**Fig. 1:**
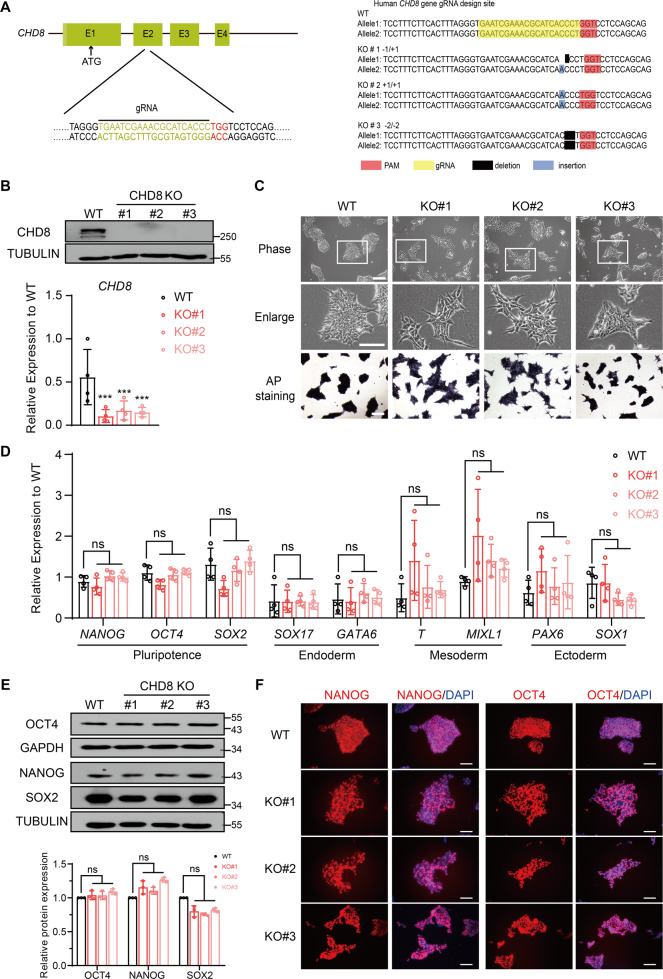
CHD8 is dispensable for pluripotency maintenance of human ESCs. **A** Left, schematic diagram showing the gRNA targeting the exon 2 (E2) of CHD8; Right, schematic diagram showing the genotype (frameshift) of the three CHD8 knockout (CHD8-KO) clones generated by Crispr/Cas9 technology. **B** Top, Western blot analysis showing expression levels of CHD8 in wildtype (WT) and CHD8-KO ESCs (*n* = 3); Bottom, RT-qPCR of *CHD8* expression in WT and CHD8-KO (*n* = 4). **C** Top, Bright-field and enlarger images showing the morphology of WT and CHD8-KO clones (KO#1, KO#2, KO#3) (*n* = 3); Bottom, Representative alkaline phosphatase staining of WT and CHD8-KO ESCs (*n* = 3). Scale bar = 200 µm. **D** RT-qPCR of representative pluripotent and lineage genes expression in WT and CHD8-KO ESCs (*n* = 4). **E** Western blot analysis showing the protein levels of pluripotent markers in WT and CHD8-KO ESCs (*n* = 3). **F** Representative immunofluorescence staining of pluripotent marker NANOG and OCT4 for WT and CHD8-KO ESCs (*n* = 3). Scale bar = 100 µm. The error bars represented the mean ± SD and the significance level was calculated by Student’s *t*-test (two-tailed, equal variance) (ns means not statistically significant, *P < 0.05, **P < 0.01, ***P < 0.001).

To understand the function of CHD8 in human ESCs and lineage differentiation, we generated CHD8 knockout ESCs by CRISPR/Cas9 technology. The guide RNA (gRNA) was designed to target the second exon of CHD8 (Fig. 1A, Left). We examined 48 colonies in total and successfully obtained three knockout clones with different genotype (KO#1, 1 bp deletion and 1 bp insertion in individual allele; KO#2, 1 bp insertion at both alleles; KO#3, 2 bp deletion at both alleles) (Fig. 1A, Right). To confirm that the CHD8 is knocked out successfully, we performed Western blot, which showed no detectable CHD8 protein in KO#1, KO#2 or KO#3 clones (Fig. 1B, Top), and quantitative reverse transcription PCR (RT-qPCR) analysis, which showed the *CHD8* RNA expression was significantly downregulated as well (Fig. 1B, Bottom). All the three knockout clones displayed relatively loose but colony morphology (Fig. 1C, Top). Since the positive staining of the alkaline phosphatase (AP) is one of the characteristics of pluripotency [25], we performed this assay to test if CHD8 knockout affected ESC pluripotency. Interestingly, our result indicated all the three knockout clones having positive AP activity (Fig. 1C, Bottom). We then assessed whether CHD8 knockout affected the expression of pluripotent markers and lineage-specific markers. The RT-qPCR of pluripotent genes (including *NANOG*, *SOX2*, and *OCT4*) and lineage-specific genes (*PAX6* and *SOX1* for ectoderm; *SOX17* and *GATA6* for endoderm; *T* and *MIXL1* for mesoderm) revealed that there was no significant difference between CHD8 knockout and wildtype ESCs (Fig. 1D). Western blot analysis also demonstrated that CHD8 knockout did not alter the protein levels of pluripotent marker OCT4, NANOG and SOX2 (Fig. 1E). Immunofluorescence staining result further confirmed that OCT4 and NANOG in the wildtype and CHD8 knockout groups were comparable (Fig. 1F). These results together suggested that CHD8 is dispensable for the pluripotency maintenance of human ESCs.

### CHD8 knockout slightly affects the apoptosis, cell cycle, and proliferation of human ESCs

**Fig. 2:**
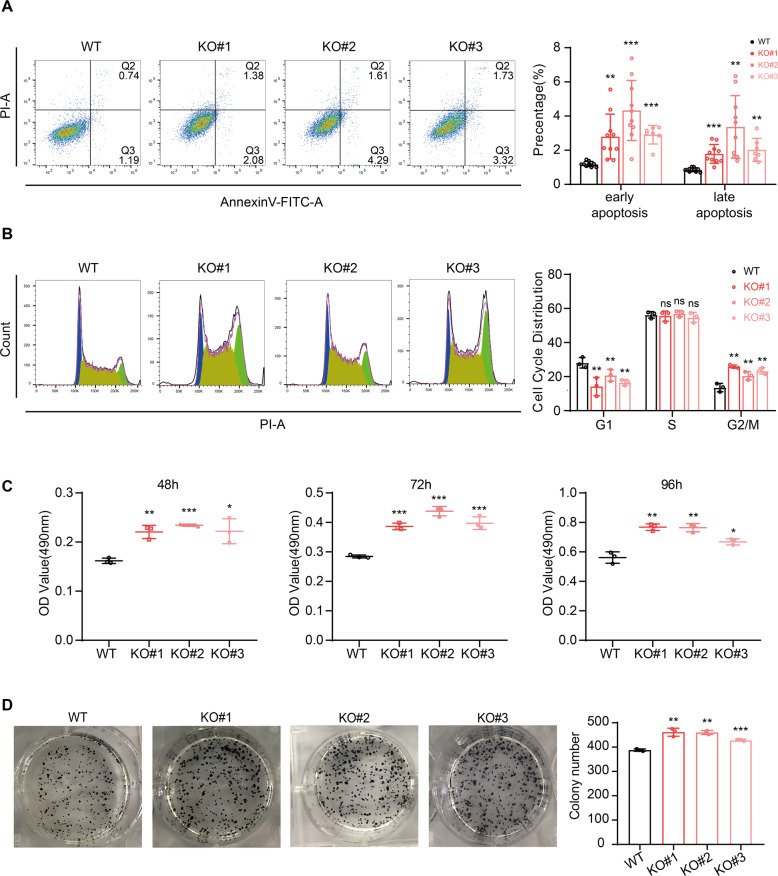
CHD8 depletion alters the apoptosis, cell cycle, and proliferation process in human ESCs. **A** Apoptosis assay with PI and AnnexinV double staining detected by FACS. The results were calculated and compared with WT ESCs (*n* = 7). **B** Cell cycle analysis using PI staining in WT and CHD8-KO ESCs. The populations of different phases were calculated in FlowJo (v10.4.0) (*n* = 3). **C** The OD490 value for MTT assay in three time points during culture (*n* = 3). **D** Colony-forming analysis visualized by AP staining and the statistics of the colonies number was shown in bar graph (*n* = 3). The error bars represented the mean ± SD and the significance level was calculated by Student’s *t*-test (two-tailed, equal variance) (ns means not statistically significant, *P < 0.05, **P < 0.01, ***P < 0.001).

Since CHD8 was reported to link with massive apoptosis resulting in early embryonic lethality [14] and required for cell cycle gene activation during G1/S transition [26], we then examined the apoptosis, cell cycle and growth rate of the CHD8 knockout ESCs. We performed the AnnexinV and propidium iodide (PI) double staining assay, in which the AnnexinV + /PI- and AnnexinV + /PI + populations represent the early and late apoptosis, respectively [27]. The results showed that the CHD8 knockout ESCs exhibited a significantly higher level of both early and late apoptosis than wildtype albeit in a low level that is less than 8% (Fig. 2A). Normally, the ESCs have a long S phase and short G1 phase, thus we performed the cell cycle analysis with the PI staining to test CHD8 knockout ESCs [28, 29]. Intriguingly, we observed a shortened G1 phase, comparable S phase and longer G2/M upon knocking out CHD8 (Fig. 2B). As shortened G1 correlates fast proliferation and longer G2/M usually correlates slow proliferation, we further performed MTT and colony formation assays, which are used to quantify cell proliferation rate [25, 30]. Indeed, the MTT result showed CHD8 knockout ESCs exhibited a higher proliferation rate than wildtype ESCs (Fig. 2C). Consistent with MTT result, the colony-forming assay also showed slight but significant higher proliferation rate in CHD8 knockout ESCs (Fig. 2D). Previous reports have shown that a shortened G1 phase induced cell proliferation [31] and apoptosis could arrest G2/M phase [32], which might explain our observation in the cell cycle and apoptosis. Thus, these results suggested that CHD8 knockout induced a slight increased apoptosis, quicker transition of G1/S and a higher proliferation rate in human ESCs.

### CHD8 knockout does not significantly influence the global transcriptome of ESCs

**Fig. 3:**
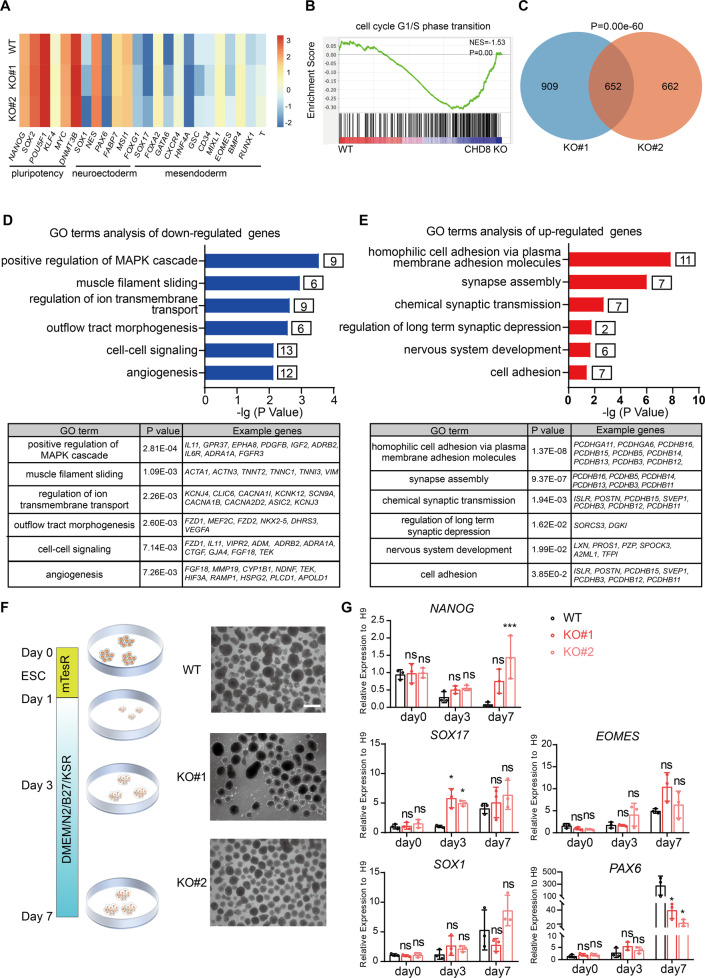
Transcriptome analysis and EB assay for CHD8-KO ESCs. **A** Heatmap showing representative genes of pluripotency and lineage specification in the RNA-seq analysis of CHD8-KO#1, KO#2 and WT ESCs. **B** The GSEA showing the cell cycle related genes in WT and CHD8-KO ESCs. **C** Venn diagram showing the number and *P-* value of overlap DEGs between KO#1 and KO#2 compared with WT. **D**, **E** GO terms of down- and up-regulated genes in CHD8-KO ESCs. **F** Schematic diagram showing the EB formation assay and bright-field images of WT and CHD8-KO EBs at day 7 (*n* = 3). **G** RT-qPCR of selected genes during EB formation (*n* = 3). The error bars represented the mean ± SD and the significance level was calculated by Student’s *t*-test (two-tailed, equal variance) (ns means not statistically significant, *P < 0.05, **P < 0.01, ***P < 0.001).

To explore whether loss-of-function of CHD8 impacted on global gene expression in ESCs, we performed RNA-seq analysis. Since there was no obvious phenotypic difference among the three CHD8 knockout cell lines, we chose two (KO#1 and KO#2) for RNA-seq and the following studies. From genome-wide transcriptome analysis of these two different CHD8 knockout cell lines, we observed a quite similar global expression pattern (Fig. S1A). Importantly, the differentially expressed genes (DEGs) compared with wildtype cells exhibited similar gene ontology (GO) terms between two CHD8 knockout lines (Fig. S1B, C and Supplementary dataset 1). Consistent with RT-qPCR results (Fig. 1C, D), no significant change on pluripotency and three germ layers-associated genes was observed (Fig. 3A). Gene set enrichment analysis (GSEA) also revealed significant enrichment in the cell cycle progress between wildtype and CHD8 knockout cells (Fig. 3B). Although these two different knockout lines have different numbers of DEGs compared with wildtype cells, there is a significant overlap between these differentially expressed genes (Fig. 3C). Thus, we conducted the following analysis using the overlapping DEGs between the two CHD8 knockout lines. The DEGs contain more downregulated genes (493/652) than upregulated (159/652) (Fig. S1D), which suggests CHD8 mainly acts as transcriptional activator. GO analysis indicated that downregulated genes were enriched in muscle filament sliding, cell-cell signaling and angiogenesis, while upregulated genes were enriched in synapse assembly (Fig. 3D, E) (Supplementary dataset 2), providing a clue that CHD8 might affect the neural development.

### CHD8 perturbs spontaneous neuroectoderm differentiation in embryoid bodies formation

To investigate whether CHD8 is indeed involved in lineage differentiation, we performed the conventional embryoid bodies (EBs) assay to ascertain the ability of spontaneous differentiation [33]. Both the CHD8 knockout and wildtype ESCs were capable of forming typical EBs without any distinguishable difference in the morphology and size (Fig. 3F). We then collected EBs at day 3 and day 7 to compare the expression levels of key genes marking pluripotency or three germ layers. The time course RT-qPCR analysis showed the pluripotent gene *NANOG* was not remarkably reduced in CHD8 knockout lines compared to wildtype during EB differentiation. Despite the endoderm and mesoderm markers such as *SOX17* and *EOMES* and neuroectoderm marker *SOX1* did not show obvious difference, the expression level of the early neuroectoderm marker *PAX6* was dramatically decreased in the CHD8 knockout EBs at day 7 (Fig. 3G). These results suggested that CHD8 perturbs spontaneous neuroectoderm differentiation and might be required for neural lineage differentiation.

### CHD8 is not required for definitive endoderm differentiation

**Fig. 4:**
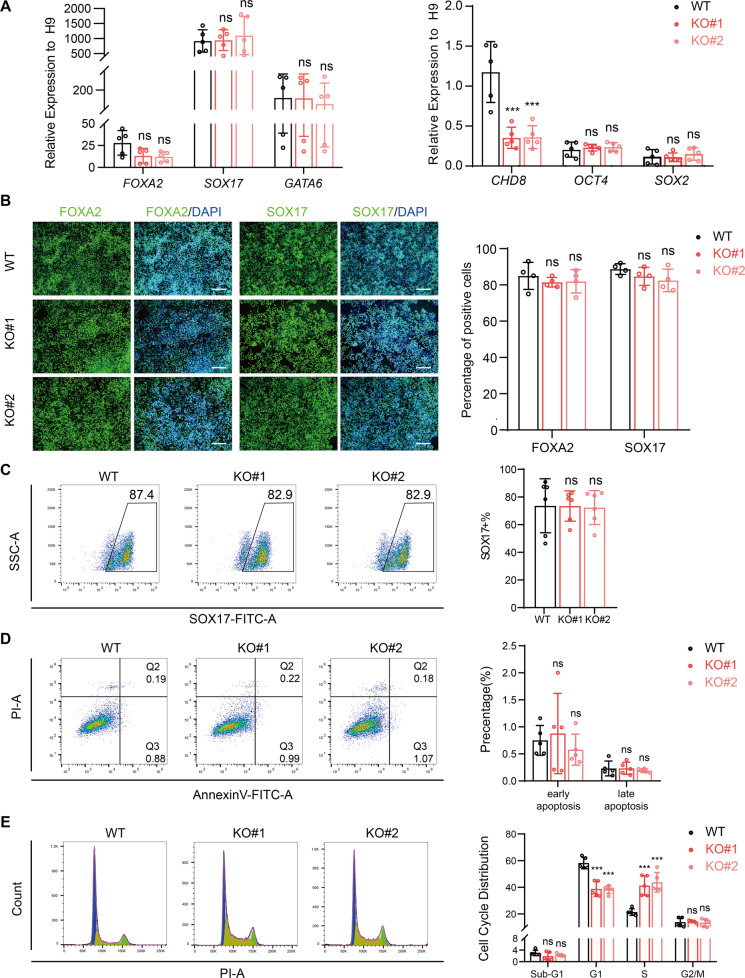
CHD8 knockout does not affect definitive endoderm differentiation. **A** Left, RT-qPCR analysis of endoderm genes *FOXA2, SOX17* and *GATA6*; Right, RT-qPCR analysis of *CHD8* and pluripotent genes *OCT4* and *SOX2* at day 3 of differentiation (*n* = 5). Wildtype ESCs at day 0 served as control. **B** Immunofluorescence of endoderm marker FOXA2 and SOX17 in WT and CHD8KO groups at day 3 of differentiation. The percentage of FOXA2- or SOX17-positive cells was calculated (*n* = 4). Scale bar = 200 µm. **C** Flow cytometric analysis showing the percentage of SOX17-positive cells in differentiated WT and CHD8-KO cells (n = 5). **D**, **E** AnnexinV/PI assay (**D**) and Cell cycle analysis using PI staining (**E**) in differentiated WT and CHD8-KO definitive endoderm cells (*n* = 5). The populations of different cell cycle phases were calculated in FlowJo (v10.4.0). The error bars represented the mean ± SD and the significance level was calculated by Student’s *t*-test (two-tailed, equal variance) (ns means not statistically significant, *P < 0.05, **P < 0.01, ***P < 0.001).

To further investigate the functional role of CHD8 in lineage determination of human ESCs, we first performed the definitive endoderm differentiation [34]. After differentiation, we analyzed the expression levels of endoderm markers (*FOXA2, SOX17*, and *GATA6*) and pluripotent markers (*OCT4* and *SOX2*) by RT-qPCR. These data showed both knockout and wildtype cells exhibited comparable expression patterns with increased endoderm markers and decreased pluripotent markers (Fig. 4A), which was consistent with the EB assay (Fig. 3F). Similar to the RT-qPCR result, the immunostaining result of SOX17 and FOXA2 and flow cytometric analysis for SOX17 also supported the conclusion that CHD8 was not required for definitive endoderm differentiation (Fig. 4B, C). Further, we also examined the apoptosis and cell cycle in differentiated endoderm cells. There was no obvious cell death in both CHD8 knockout and wildtype group (Fig. 4D). However, the cell cycle analysis indicated CHD8 knockout led to reduced G1 phase but increased S phase with no distinguishable G2/M phase (Fig. 4E). Taken together, our results indicate CHD8 has no significant role on endoderm differentiation efficiency and cell apoptosis during endoderm differentiation.

### CHD8 knockout disrupts early neuroectoderm differentiation

**Fig. 5:**
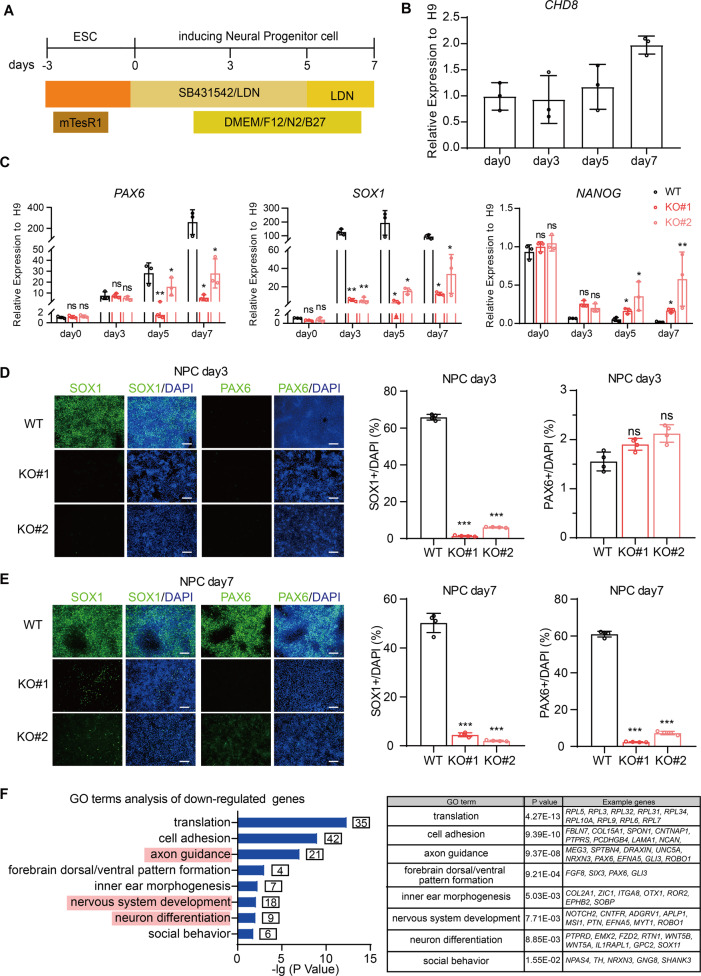
CHD8 loss-of-function disrupts neuroectoderm differentiation. **A** Cartoon schematic showing the protocol of differentiating human ESCs toward neural fate. **B** the dynamic gene expression of *CHD8* during neuroectoderm differentiation (*n* = 3). **C** RT-qPCR analysis of neuroectoderm genes *PAX6* and *SOX1* and pluripotent gene *NANOG* at day 0, 3, 5, 7 of differentiation (*n* = 3). Wildtype ESCs at day 0 served as control. **D**, **E** Immunofluorescence of neuroectoderm markers SOX1 and PAX6 in wildtype and knockout groups at day 3 (**D**) and day 7 (**E**) of differentiation. The percentage of SOX1- or PAX6-positive cells was calculated (*n* = 4). Scale bar = 200 µm. **F** GO enrichment analysis for downregulated genes in CHD8 knockout NPC (*P* < 0.05, log_2_(fold-change) < −1). The error bars represented the mean ± SD and the significance level was calculated by Student’s *t*-test (two-tailed, equal variance) (ns means not statistically significant, *P < 0.05, **P < 0.01, ***P < 0.001).

Next, we used a well-established protocol to induce CHD8 knockout and wildtype ESCs towards neural progenitor cell (NPC) stage with minor modifications [35] (Fig. 5A). *CHD8* is universally expressed during entire neuroectoderm differentiation with the highest expression at day 7 when achieving complete differentiation (Fig. 5B). The time course RT-qPCR results showed that the neural progenitor marker *PAX6* was gradually increased over time and had a 200-fold increase at day 7 compared with ESCs in wildtype cell line; the other marker *SOX1* was abundantly expressed from day 3 and maintained high level during neural progenitor differentiation with continuous declination of pluripotent marker *NANOG* (Fig. 5C). The immunostaining of PAX6 and SOX1 at day 3 and day 7 also revealed SOX1-positive cells appeared and maintained from day 3 and PAX6-positive cells emerged at day 7 in wildtype cell line (Fig. 5D, E). These results suggested that our neuroectoderm conversion of human ESCs was robust and highly efficient. Using the same differentiation condition, we found CHD8 knockout ESCs exhibited severe defect in differentiation of neural progenitors. The RT-qPCR results showed that the expression of neural progenitor marker *PAX6* and *SOX1* were dramatically reduced in CHD8 knockout groups compared to wildtype group, and the pluripotent gene *NANOG* did not decreased in CHD8 knockout groups during differentiation (Fig. 5C). Moreover, by immunofluorescence staining, we observed that the signal of SOX1 or PAX6 was dramatically decreased at the day 3 and day 7 in CHD8 knockout groups (Fig. 5D, E). These results indicated that CHD8 knockout disrupted the neuroectoderm differentiation capacity of human ESCs.

**Fig. 6:**
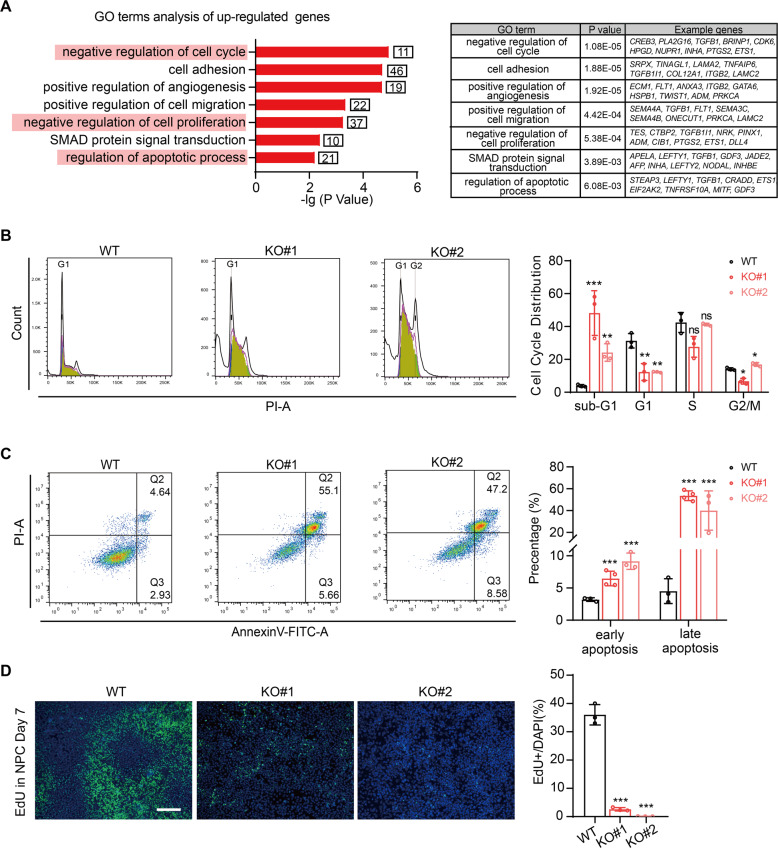
CHD8 knockout induces dramatic apoptosis in neuroectoderm progenitor stage. **A** GO analysis of upregulated genes in CHD8 knockout neuroectoderm progenitors (log_2_(fold-change) > 1, *P* < 0.05). **B** Cell cycle analysis using PI staining in wildtype and CHD8-KO neuroectoderm progenitors by FACS. The populations of different phases were calculated in FlowJo (v10.4.0) (*n* = 3). **C** AnnexinV/PI assay in wildtype and CHD8-KO neuroectoderm progenitors by FACS (*n* = 3). **D** Representative EdU staining image of wildtype and CHD8-KO neuroectoderm progenitors (*n* = 3). Scale bar = 200 µm. The error bars represented the mean ± SD and the significance level was calculated by Student’s *t*-test (two-tailed, equal variance) (ns means not statistically significant, *P < 0.05, **P < 0.01, ***P < 0.001).

We further performed a transcriptome profiling by using the cells at day 7 of differentiation to examine the effect of CHD8 knockout on neuroectoderm differentiation, and conducted the following analysis using the overlapping differentially expressed genes between the two CHD8 knockout lines compared to wildtype cells (Fig S2 and Supplementary dataset 1 and 2). The RNA-seq analysis revealed that the downregulated genes due to CHD8 depletion were highly enriched in terms associated with neuronal function and nervous system (Fig. 5F), while the upregulated genes were enriched in terms involved in SMAD signal, which negatively regulates neural differentiation (Fig. 6A). These results elucidated that CHD8 knockout indeed disrupted the gene regulation network of neuroectoderm.

### CHD8 knockout induces massive apoptosis during neuroectoderm differentiation

Since deletion of CHD8 alters expression of the genes that involved in cell cycle, apoptosis process and proliferation (Fig. 6A), we next examined the apoptosis and cell cycle status in neuroectoderm stage within CHD8 knockout cells. The cell cycle analysis indicated a significant portion of CHD8 knockout cells paused at sub-G1 stage (Fig. 6B), which suggested that the CHD8 knockout cells may undergo apoptosis [36]. Further, we performed apoptosis assay using AnnexinV-FITC and PI double staining, and the result showed the CHD8 knockout cells indeed exhibited much higher apoptosis level than wildtype, of which 50% cells undergoing late apoptosis at the day 7 of neuroectoderm differentiation (Fig. 6C). In addition, we employed EdU staining to explore the role of CHD8 in proliferation during neuroectoderm differentiation [37], and the result showed nearly no EdU-positive cells in CHD8 knockout cells at the day 7 of neuroectoderm differentiation, while in wildtype neuroectoderm cells there was a high proliferation with 40% EdU-positive cells (Fig. 6D). In summary, CHD8 deficiency results in aberrant apoptosis and terminates cell proliferation during neuroectoderm differentiation.

### CHD8 is required for activation of neurogenesis genes and repression of apoptosis-related genes during neuroectoderm differentiation

**Fig. 7:**
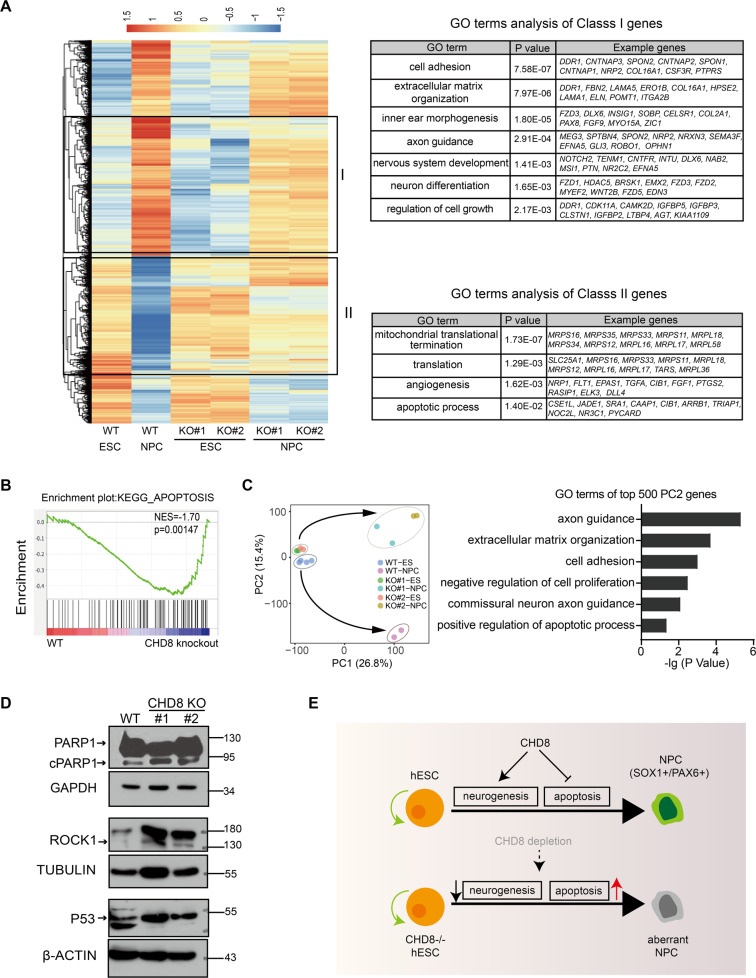
CHD8-KO blocks the activation of neural genes and results in derepression of apoptosis-related genes during neuroectoderm differentiation. **A** Heatmap showing CHD8-KO affected genes during neuroectoderm differentiation. Go terms of these genes (i.e., class I and class II genes) were also shown. **B** The GSEA showing the expression pattern of apoptosis-related genes in WT and CHD8-KO NPCs. **C** The PCA correlation map of WT and CHD8-KO in ESCs and NPCs and the GO terms of top PC2 genes. **D** Western blot analysis showing the PARP1, ROCK1, and P53 protein expression in WT and CHD8-KO NPCs. cPARP1 represents the cleaved PARP1. **E** The cartoon showing the different roles of CHD8 in ESCs and NPCs.

To further understand how CHD8 knockout blocked the neuroectoderm differentiation and resulted in severe cell death in NPC stage, we performed a comprehensive transcriptome analysis. Many genes are activated during neural differentiation, and the majority of these genes fails to be activated or are significantly reduced their expression in the two CHD8 knockout lines (Fig. 7A). Since we are interested in the function of CHD8, we mainly focused on the genes that should be activated but reduced their expression in CHD8 knockout (here referred to Class I), and found these genes are enriched in neural lineage determination, consistent with the phenotype of defected neuroectoderm differentiation. In addition, the genes that should be repressed in NPCs but still expressed in the CHD8 knockout (here referred to Class II) are enriched in angiogenesis and apoptosis (Fig. 7A). Consistent with the GO analysis of Class II, the GSEA (Gene set enrichment analysis) confirmed the dysregulation of apoptosis-related genes (Fig. 7B) in CHD8 knockout NPC, which might contribute to the observed massive apoptosis in NPC stage upon CHD8 knockout (Fig. 6C). In addition, the principal component analysis (PCA) showed that CHD8 knockout and wildtype ESCs clustered together, but the NPCs pointed to different orientation along with PC2, which also represented the terms of cell proliferation and apoptosis (Fig. 7C). We further examined the protein levels of some key apoptosis-related genes and found cleaved PARP1 (cPARP1), ROCK1 and P53 maintained the higher levels in CHD8 knockout NPCs (Fig. 7D). Altogether, these results suggested that loss of CHD8 leads to apoptosis-related genes extremely active during neuroectoderm differentiation.

## Discussion

In this study, we generated CHD8 knockout human ESCs and subjected these knockout cells to definitive endoderm and neuroectoderm differentiation to dissect the role of CHD8 in pluripotency maintenance and lineage differentiation. We found CHD8 was not essential for pluripotency of human ESCs and definitive endoderm differentiation but absolutely required for neuroectoderm differentiation. CHD8 knockout resulted in severe defect of activation of neural lineage genes that not only explained the blocked neuroectoderm differentiation, but also caused significant derepression of apoptosis-related genes during neurogenesis, which led to massive cell death in NPC stage (Fig. 7E).

CHD8 belongs to chromodomain-helicase DNA-binding (CHD) family proteins, which act as chromatin remodelers functioning in the regulation of stem cells maintenance and differentiation [3, 38, 39]. Using the CHD8 knockout human ESCs, we found the complete depletion of CHD8 has no significant effect on the expression of pluripotent markers, however, the ESCs with CHD8 knockout exhibit altered cell cycle progress with a shortened G1 phase, eventually resulting in modestly promoted cell proliferation (Fig. 2). Similarly, the *Chd9* knockdown mouse ESCs also show a modestly shortened G1 phase and promoted cell proliferation [40]. However, *Chd1*, *Chd2* or *Chd7* seems not to regulate the cell cycle or proliferation, but both *Chd1* and *Chd2* are required for the proper expression of pluripotent genes in mouse ESCs [38, 39, 41, 42]. In addition, CHD8 knockout results in severe failure of activation of neural lineage genes and induced apoptosis-related genes leading to the massive cell death during neurogenesis (Fig. 7A, B), which contribute to the blocked neuroectoderm differentiation. In fact, several CHD family members have been reported to participate in neurogenesis and the related mutations in these CHD members are correlated with neurodevelopmental disorders [6]. CHD2 knockout causes impaired cortical interneuron differentiation from human ESCs or failure of the neural differentiation from mouse ESCs, while *Chd2* knockdown in E13.5 mouse cortices mediated by siRNA leads to reduced proliferation and premature differentiation of the progenitor cells [3, 42, 43]; CHD3, CHD4 and CHD5, as a component of nucleosome remodeling and deacetylase (NuRD) complex, are required for proliferation and migration of neuron in cortical development shown by mouse knockdown experiments [44, 45]; human ESCs with *CHD7* knockdown fail to differentiate into neural crest-like cells, and loss of *Chd7* in mouse ESCs impaired the neuronal lineage differentiation [46, 47]. Additionally, CHD1 knockout in mouse and human ESC models leads to a high propensity for neuronal differentiation at the expense of mesendoderm differentiation [38, 41]. These reports together indicate that the CHD family have a general and critical role in neurogenesis and/or cell cycle control although individual member has diverse function as well.

*CHD8* is reported as one of the most high-risk mutated genes in ASD and plays a vital role in neuronal differentiation. In the previous studies, the role of *CHD8* in ASD pathophysiology had been explored with various models using haploinsufficient models (*CHD8* knockdown and heterozygous knockout) [20, 21, 23, 24], in which neurectoderm and the further neuronal differentiation were successfully achieved. In our work, we generated CHD8 knockout human ESCs and these homozygous knockout ESCs showed severe defect in neuroectoderm differentiation. These observations based on different models provide a comprehensive understanding of CHD8 function in different biological processes including pluripotency maintenance, early neural progenitor differentiation and neuronal specification. In addition, one characteristic of *CHD8* mutant in ASD is macrocephaly [48]. Our study showed CHD8 knockout induced cell apoptosis and inhibited proliferation during neuroectoderm differentiation. This result seems contrast with the *CHD8* mutants in ASD leading to macrocephaly. While the mouse model of the *Chd8* heterozygous (half decreased of *Chd8*) displayed macrocephaly with increase in proliferation of NPCs [49], but very interestingly, *in utero Chd8* knockdown (nearly 70% decreased of *Chd8*) resulted in impaired proliferation of NPCs [15]. Dosage might contribute to the difference and these reports suggest the complete depletion of CHD8 may cause worse phonotype like in mouse that induced abundant apoptosis and embryonic lethal [14]. Additionally, because our experiments are limited to analysis of neuroectoderm, we cannot elucidate the role of CHD8 in development of other cell types. It is possible that deletion of CHD8 in other cells could alter the size or number of these cells, contributing to the macrocephaly phenotype observed among people with ASD carrying *CHD8* mutations. Another possible reason is that CHD8 may paly different functions in different stages during neurogenesis. Supporting this notion, here we found that CHD8 play diverse functions in ESC stage and neuroectoderm differentiation.

In the clinic, heterozygous *CHD8* mutations rather than homozygous are identified in ASD patients. One possibility might be the homozygous mutations in CHD8 may cause more severe defect and result in lethality, which is supported by the CHD8 knockout mouse study [14]. Nevertheless, whether the CHD8 knockout human ESC and differentiation model could affect ASD-risk genes and ASD-related pathway like other CHD8 model [23] is an interesting question. We noticed that Wang and colleagues constructed a heterozygous CHD8 human ESC line (CHD8^+/-^) and performed neural differentiation, from which we downloaded the RNA-seq data in NPC (no data available in ESCs) [23]. We obtained their differentially expressed genes by using the parameters (fold-change > 2 and *P*-value < 0.05) and performed the correlation analysis with our data (the *P*-value was calculated by the TBtools [50]). There is a significant overlap between the differentially expressed genes from Wang’s data and our data (*P* = 1.91 × 10^−16^); moreover, the overlapped genes are significantly enriched in the terms of “cell adhesin” and “forebrain neuron differentiation” (Fig. S3A). We also observed the similar correlation between our data and the report based on CHD8 knockout mouse ESC-derived NPCs [51] (Fig. S3B). In addition, we collected more transcriptomics data of the CHD8 knockdown or hetero/homozygous form the published literatures including human and mouse ESC differentiation or primary neural progenitor cell line studies [15, 21, 23, 24, 51, 52] and calculated their differentially expressed genes. We then compared all other CHD8 models with our transcriptomics data in NPC to determine the similarity. From this comprehensive analysis, the data from Wang’s heterozygous CHD8 knockout in ESC-derived NPC and neuron stage indeed exhibited mostly significant enrichment with our data (Fig. S3C) (*P* = 1.91 × 10^−16^, Odd ratio (OR) = 2.39; and *P* = 4.49 × 10^−17^, OR = 1.82, respectively). Next, we collected the ASD gene sets from public database (SFARI, AustimKb2.0) and individual reports [53–57] and compared the expression pattern with our knockout model. While the CHD8-affected genes in ESC stage did not show enrichment with these ASD gene sets (Fig. S3D), the CHD8-affected genes in NPC stage showed significant enrichment with the AustimKb2.0, SFARI, Iossifov’s and De Rubeis gene sets (Fig. S3E), indicating the homozygous CHD8 knockout indeed influenced the ASD-related genes during neurogenesis rather than in ESCs. Moreover, we also found a few pathways involved in ASD, such as the synaptic transmission, translation and social behavior [54, 55] in our data (Figs. 3E and 5F), Taken together, CHD8 controls neuroectoderm differentiation and complete knockout of CHD8 indeed affects ASD-risk genes and pathways.

CHD8 is an ATP-dependent chromatin remodeler and plays an important role in regulation of gene transcription [7]. In most cases CHD8 acts as a transcription activator since CHD8 is interacting with H3K4me2 and required for RNAPII recruiting to the enhancers [58, 59]. In our data, loss-of-function of CHD8 led to 2-fold more downregulated genes than upregulated genes in ESCs; moreover, early neuroectodermal genes failed to be activated during neural differentiation upon CHD8 knockout (Fig. 7A). These data supported that CHD8 contributed to gene activation both in ESCs and neuroectoderm differentiation. On the other hand, CHD8 interacts with CTCF and inhibits gene expression via a CTCF-dependent manner [60]. A previous report showed that in HEK-293T cells CHD8 recruited the histone H1 to suppress p53-mediated apoptosis. More importantly, knockdown p53 in CHD8 knockout mouse embryo could partially delay the lethal time to E10.5 [14, 61]. Similar results were found in CHD8 knockout mouse ESCs in which CHD8 interfered the accessibility of loci containing *p53/Ctcf* binding motif [51]. In our study, we observed that a significant portion of genes, including apoptosis-related genes, failed to be repressed during neurogenesis (Fig. 7A, B). Our data together supported that CHD8 might act as both transcriptional activator and repressor in human neurogenesis, however, due to the lack of antibody workable for ChIP analysis, the detailed mechanism is awaiting further dissection.

In summary, our current study illustrated the importance of CHD8 in human early neuroectoderm differentiation by constructing human ESCs with complete loss-of-function of CHD8. CHD8 is necessary for the activation of neural lineage specifiers during neurogenesis, and meanwhile, CHD8 prevents the differentiated neural progenitor cells from apoptosis. This study facilitates our comprehensive understanding on CHD8 in human ESCs and early neuroectoderm differentiation and provides new insights into CHD8 function.

## Methods and materials

### Cell culture

Human embryonic stem cell H9 (also WA09) was cultured on plates coated with Matrigel (Corning, USA, Cat#354277) in mTesR1 (STEMCELL Technologies, USA, Cat#85850). These cells were maintained at 37 °C with 5% CO2 in cell incubator and passaged by Accutase (STEMCELL Technologies, USA, Cat#07922) usually every 4 days. The medium was replaced every day.

### Generation of CHD8 knockout human ESC

CRISPR/Cas9 genome editing technology was used to generate CHD8 knockout human ESC. Briefly, a single gRNA was designed by targeting the exon 2 (near to start codon) of CHD8 gene on the website (http://crispr.mit.edu/). The guide sequence (5’-TGAATCGAAACGCATCACCC-3’) was cloned into the pX459 expression vector. To knock out the CHD8 gene, 1 million cells were electroporated with 5 μg pX459 plasmid containing guide sequence and then replated onto Matrigel-coated 6-well plate in mTesR1 medium with 10 μM Y-27632 (Selleck, USA, Cat#S1049). After 24 h culture, the puromycin (1 μg/ml) (Santa Cruz Biotechnology, USA, Cat#58582) was added for 48 h to select positive cells. By gradual dilution strategy to colonize the survived cells, we extracted DNA for genotyping by sanger sequencing. We used western blotting to further validate the positive clones.

### Alkaline phosphatase (AP) staining

In all, 2 × 10^4^ single ESCs were seeded per well in 24-well plate and were grown for 3 days. The alkaline phosphatase detection kit (Beyotime, China, Cat#C3206) was used for this assay according to the instruction. Briefly, cells were fixed with 4% paraformaldehyde at room temperature for 20 min, followed by incubated with staining mixture in dark overnight at room temperature. Colonies were visualized and photographed using microscopy. AP staining was also used to measure the numbers of pluripotent colonies in colony-forming assay.

### MTT (3-(4,5-dimethylthiazol-2-yl)-2,5-diphenyltetrazolium bromide) assay

Cells were dissociated into single cells by Accutase and seeded with the density of 1.5 × 10^3^ cells per well in 96-well plate. When culturing at the 48, 72, and 96 h, 10% MTT was added into culture medium for another 4 h incubation at 37 °C incubator. Then the MTT-medium was removed and 200 μl DMSO (dimethyl sulfoxide) was added into each well for another 10 min at 37 °C incubator. Last, the plate was shaking on an orbital shaker for 10 min and the absorbance value was measured at 490 nm with MD SpectraMax i3x (Molecular Devices, USA).

### Cell cycle and apoptosis analyses

For cell cycle assay, a cell cycle detection kit (Keygen, China, Cat#KGA512) was used according to the protocol included in the kit. In brief, cells were dissociated into single cells, and about 1 million cells were fixed in 75% ethanol at 4 °C overnight. After aspirating the fixation solution and washing the cells once with DPBS, 500 μl DPBS with 100 μg/ml RNase A was added to resuspend the cell for incubation at 37 °C for 30 min. Then the cells were added propidium iodide (PI) with final concentration of 50 μg/ml and incubated at room temperature for another 30 min in dark. Finally, a FACSCelesta flow cytometer (BD Biosciences, USA) was used to analyze these cells. Then these sorted cells were divided into each cell cycle phase (subG1, G1, S, and G2/M) based on the PI intensity in the FlowJo (v10.4.0) and calculated the proportion of each phase.

An AnnexinV-FITC/PI cell apoptosis detection kit (Keygen, China, Cat#KGA106) was used for apoptosis analysis. About 1 × 10^5^ single cells were resuspended by binding buffer supplemented with AnnexinV-FITC and PI at room temperature for 15 min. Finally, a FACSCelesta flow cytometer was used to analyze these cells.

### Embryoid body (EB) formation

EB formation was performed according to a recent report [33]. In brief, 5 × 10^5^ ESCs were resuspend in mTesR1 medium with 10 μm Y-27632 and then plated on 6-well low attachment plates at 37 °C with 5% CO_2_ incubator to allow cells self-aggregation. After 24 h, the small EBs were transferred to a new low attachment plate and these aggregates were cultured in 3 ml EB medium (DMEM containing 20% knockout serum replacement (KSR, Gibco, USA, Cat#10828028), 1x glutamine supplement (Gibco, USA, Cat#35050079), 1% NEAA (Gibco, USA, Cat#11140050), and 0.2% β-Mercaptoethanol (Gibco, USA, Cat#21985023)). The EB medium was changed every 2 days. On day 7, the EBs were collected for gene expression analysis.

### EdU proliferation assay

To investigate the cell proliferation rate, we performed an EdU assay with a KFlour488 Click-iT EdU imaging detection kit (Keygen, China, Cat#KGA331-100) according to the instructions. In brief, cells were incubated with 10 µM EdU and then washed and fixed with 4% paraformaldehyde, followed by glycine buffer for neutralization. After permeabilized in 0.5% Triton-100 in DPBS, 200 μl EdU-kFlour488 Click-iT staining mix was added to each well and incubated for 30 min in dark. The cells were then washed and stained with 5 μg/ml Hoechst 33342 for 10 min in dark. Cells were visualized and counted using fluorescence microscopy.

### Neuroectoderm differentiation

To induce neural differentiation, wildtype or CHD8 knockout human ESCs were seeded onto Matrigel-coated 24-well plates at the density of 5 × 10^4^ cells. After 3 days’ culture with mTeRS1 medium to achieve a 95% confluence, the neuroectoderm differentiation was initiated by neural induction medium (DMEM/F12 (Gibco, USA, Cat#C11995500BT), 0.5× N2 (BasalMedia, China, Cat#S430J4)), 0.5× B27 (BasalMedia, China, Cat#S441J7), 1% Gluta-max, 1% NEAA, 1x penicillin/streptomycin (Gibco, USA, Cat#10378016), 0.1% β-Mercaptoethanol) plus 10 μM SB431542 (Selleck, USA, Cat#S1067), and 0.1 μM LDN193189 (Selleck, UAS, Cat#7507). After 5 days of neural induction, the TGF-β inhibitor SB431542 was withdrawn from the neural induction medium for another 2 days’ culture. The medium was changed every day. The cells were collected for immunofluorescence assay at day 3 and 7, and for qPCR assay at day 0, 3, 5, and 7.

### Definitive endoderm cell differentiation

To initiate definitive endoderm differentiation, wildtype or CHD8 knockout human ESCs were seeded onto Matrigel-coated 24-well plates at the density of 5 × 10^4^ cells per well with mTeRS1 medium. After 24 h, the differentiation was induced by 100 ng/ml Activin A (PeproTech, USA, Cat# 120-14 P) and 2.5 μM CHIR99021 (Selleck, USA, Cat#S2924) in the medium DMEM (Gibco, USA, Cat#C11995500BT) plus 0.5× B27 (BasalMedia, China, Cat#S441J7), 1x penicillin/streptomycin (Gibco, USA, Cat#10378016) and 0.2% BSA (YEASEN, China, Cat#B57370) for 1 day. Then the CHIR99021 was withdrawn from the induction medium for another 2 days’ culture. The medium was changed every day. The cells were collected at day 3 for further analysis.

### Flow cytometry

The culture cells were collected by Accutase and resuspended and washed twice in DPSB with 2% FBS (Gibco, USA, Cat#10100147). Then the cells were fixed with Fix/Perm Buffer for 1 h at 4 °C. After washing twice with Perm Buffer, the cells were incubated with diluted antibody (Alexa Fluor® 488-conjugated mouse anti-human SOX17 (BD, USA, Cat#562205)) for 45 min at 4 °C. Then the cells were washed and resuspended in 200 μl DPBS. Finally, a FACSCelesta flow cytometer was used to analyze these cells.

### Western blot

Undifferentiated ESCs and differentiated neuroectoderm cells were collected by dissociation and centrifuging. After once wash with DPBS, cells were lysed on ice in RIPA buffer (50 mM pH 7.4 Tris-HCl, 150 mM NaCl, 0.1% SDS, 1% NP40, 0.5% sodium deoxycholate) plus 1x cOmplete Protease Inhibitor (Roche, Switzerland, Cat#04693116001) for 1 h. Then the lysates were centrifuged at 10,000 × *g* for 10 min at 4 °C to obtain the soluble whole-protein extract. The protein concentration was quantified by the BCA protein assay kit (Thermo Fisher Science, USA, Cat#A53225) according to the manufacture’s recommendations. About 30 μg whole proteins were subjected to 6% SDS-PAGE (for CHD8) or 10% SDS-PAGE (for other proteins) according to the protein molecular mass. After transferred to a nitrocellulose membrane, 5% (w/v) BSA was used for block for 2 h at room temperature. Then the membrane was incubated overnight at 4 °C or 2 h at room temperature with the primary antibodies. After washing three times with TBST for 10 min per time, the membranes were incubated with HRP-conjugated second antibodies for 1 h at room temperature. After washing for three times with TBST for 10 min per time, the membranes were incubated with ECL for 3 min in dark and visualized using film image system in dark room. The antibodies used in present work include: CHD8 (1:500, CST, USA, Cat#11891 S), GAPDH (1:5000, Proteintech, USA, Cat#60004-l-lg), α-Tubulin (1:5000, Proteintech, USA, Cat#11224-1-AP), OCT4 (1:1000, Santa Cruz Biotechnology, USA, Cat#sc-5279), SOX2 (1:100, Santa Cruz Biotechnology, USA, Cat#sc-20088), NANOG (1:1000, Santa Cruz Biotechnology, USA, Cat#sc-33759), PARP1 (1:3000, Proteintech, USA, Cat#13371-1-AP), ROCK1 (1:1000, Proteintech, USA, Cat#21850-1), P53 (1:1000, ABclonal, USA, Cat#A19585).

### Immunofluorescence assay

The cells were fixed with 4% paraformaldehyde at room temperature for 20 min. After washing twice with DPBS, cells were blocked and permeabilized in blocking solution (DPBS with 10%(v/v) donkey serum and 0.3% triton-100) for 30 min. Then the cells were incubated with primary antibodies overnight at 4 °C. After washing three times with DPBS, cells were stained with secondary antibodies for 2 h in dark at room temperature. Then the cells were washed three times with DPBS and stained with 5 μg/ml DAPI for 10 min in dark at room temperature. Cells were visualized using fluorescence microscopy and counted used ImageJ. The antibodies for immunofluorescence assay used in present work include: PAX6 (1:200, Biolegend, USA, Cat#l901301), SOX1 (1:200, CST, USA, Cat#4195S), OCT4 (1:200, Santa Cruz biotechnology, USA, Cat#sc-5279), NANOG (1:200, CST, USA, Cat#4903), FOXA2 (HuaBio, China, Cat#ET1703-76), SOX17 (R&D Systems, USA, Cat# AF1924).

### Real-time qPCR

Total RNA was extracted from cells with HiPure Total RNA Mini Kit (Magen, China, Cat#R4111-03). Two micrograms of RNA were reverse transcribed into cDNA using the ABScript II RT Master Mix (ABclonal, USA, Cat#RK20402). Then real-time PCR was performed with 2x SYBR Green qPCR Master Mix (Bimake, USA, Cat#B21203) and run on a C1000 Touch Thermal Cycler machine (Bio-Rad, Hercules, California, USA). All Real-Time qPCR experiments were carried out at least three replicates. The relative gene expression was normalized to *GAPDH* based on the delta Ct method. The primer sequences are listed as below: *GAPDH*, AATGAAGGGGTCATTGATGG and AAGGTGAAGGTCGGAGTCAA; *NANOG*, F-CCCCAGCCTTTACTCTTCCTA and CCAGGTTGAATTGTTCCAGGTC; *OCT4*, F-CAAAGCAGAAACCCTCGTGC and TCTCACTCGGTTCTCGATACTG; *SOX2*, F-GTCATTTGCTGTGGGTGATG and AGAAAAACGAGGGAAATGGG; *PAX6*, TCCGTTGGAACTGATGGAGT and GTTGGTATCCGGGGACTTC; *SOX1*, ATTATTTTGCCCGTTTTCCC and TCAAGGAAACACAATCGCTG; *SOX17*, GCATGACTCCGGTGTGAATCT and TCACACGTCAGGATAGTTGCAGT; *EOMES*, CACATTGTAGTGGGCAGTGG and CGCCACCAAACTGAGATGAT; *GATA6*, AGTTCCTACGCTTCGCATCCCTTC and TGAACAGCAGCAAGTCCTCCCA; *T*, TATGAGCCTCGAATCCACATAGT and TATGAGCCTCGAATCCACATAGT; *MIXL1*, GAGACTTGGCACGCCTGT and GGTACCCCGACATCCACTT; *CHD8*, AAGCAAATCGGATTGTAGCAGA and AAGCAAATCGGATTGTAGCAGA; *FOXA2*, GGAGCAGCTACTATGCAGAGC and CGTGTTCATGCCGTTCATCC; *SOX17*, GCATGACTCCGGTGTGAATCT and TCACACGTCAGGATAGTTGCAGT.

### RNA-seq and data analysis

Undifferentiated ESCs and differentiated neuroectoderm cells were collected and then the total RNA was extracted according to the manufacturer’s recommendations. After quantified by a DNA/Protein Analyzer (QuaWell, Sunnyvale, California, USA), the RNAs were sent to Geekgene (Beijing, China) for RNA-seq library construction and sequencing. For data analysis, reads were qualitied by FastQC (v0.11.9) and the adapter was trimmed by Trim_galore (v0.6.2). Then these reads were aligned to the human reference genome GRCh38 using HISAT2 (v2.1.0), and gene expression counts were determined by featureCounts (v1.6.4). To determine differential expression genes, the low abundance genes with low counts (rowSums < 40) were firstly discarded and then the remaining genes were conducted with DESeq2 (v1.20.0) to define significant differences genes by setting *P*-value < 0.05 and log_2_(fold-change) > 1. After obtaining the significant differences genes, gene ontology analysis was performed on DAVID ((https://david.ncifcrf.gov/) and the heatmap was made by pheatmap (v1.0.12). The GSEA was performed on GSEA_4.1.0 software.

### Compare to other CHD8 models and ASD-risk gene sets

To explore our CHD8 KO transcriptomics datasets whether consists with other CHD8 models, we obtained their RNA-seq data from the Gene Expression Omnibus (GEO) and the information is listed in Data availability statement. The DEGs were also filtered out the non-coding genes for enrichment analysis. The fisher exact test with one tailed was used to calculate the *P-*value. The ASD-risk gene sets were obtained from the published datasets or research articles. There were two datasets, SFARI Gene 2.0 (https://gene.sfari.org/autdb/Welcome.do) [53] and AustimKB2 (http://db.cbi.pku.edu.cn/autismkb_v2) [57]. To highlight the ASD risk gene sets, we selected the SFARI set with the genes being reported >10 times (559 genes left) and the AustimKB2 with the gene having scores >16 (218 genes left). In addition, individual reports have been considered: Willsey and colleagues identifying high-confidence and probable ASD genes (referred to Wisslsy_ASD) [56], and Iossifov et al. and DeRuBeis et al. developing high-confidence ASD-risk lists form whole-exome sequencing (referred to Iossifov_ASD and DeRubeis_ASD, respectively) [54, 55]. These gene sets were used for GSEA.

### Statistical methods

All experiments were performed at least three independent replicates. Data was shown as mean ± SD. The significance level was calculated by Student’s *t-*test (two-tailed, equal variance) in GraphPad Prism 8 and *P*-values are shown with *P < 0.05, **P < 0.01, ***P < 0.001.

## Supplementary information

Supplemental material

Original data western blot

dataset 1

dataset 2

## Data Availability

The RNA-seq of other CHD8 model were obtained from the Gene Expression Omnibus and the DOI for these paper list below: Sugathan et al.: 10.1073/pnas.1405266111; Durak et al.: 10.1038/nn.4400; Wang et al.: 10.1186/s13229-015-0048-6; Wang et al.: 10.1186/s13229-017-0124-1; Sood et al.: 10.1073/pnas.1921963117 and Wilkinson et al.: 10.1038/tp.2015.62. All the RNA-seq raw data from our study have been submitted to the Gene Expression Omnibus and the accession number for this work is GSE171869.
