# Supplementary material for: CHD8 safeguards early neuroectoderm differentiation in human ESCs and protects from apoptosis during neurogenesis
Source: Cell Death Dis. 2021 Oct 22;12(11):981. doi: 10.1038/s41419-021-04292-5 (PMC8536677; doi:10.1038/s41419-021-04292-5)
Supplement: Supplementary file 1 — Supplemental material [file 41419_2021_4292_MOESM1_ESM.pdf]

CHD8 safeguards early neuroectoderm differentiation in human ESCs and protects from apoptosis during neurogenesis

Song Ding[1, #], Xianchun Lan[1, #], Yajing Meng[1], Chenchao Yan[1], Mao Li[1], Xiang Li[2, *], Jian Chen[3,4,*], Wei Jiang[1,5,6,*]

This Supplementary material consists of three Supplementary Figures and two Supplementary datasets.

Figure S1. Two different CHD8-knockout clones display similar transcriptome in ESC stage.

Figure S2. Two different CHD8-knockout clones display similar transcriptome in NPC stage.

Figure S3. Comparison of expression patterns between CHD8-KO and other CHD8 haploinsufficient models and the ASD-risk gene sets.

Supplementary dataset 1. The spreadsheets of the enriched GO terms ($p<0.1$) of up-regulated or down-regulated DEGs between individual KO and WT at ESC and NPC stage respectively.

Supplementary dataset 2. The spreadsheets of the enriched GO terms ($p<0.1$) of the overlapping up-regulated or down-regulated DEGs in the two KO clones compared with WT at ESC and NPC stage respectively.

Figure S1. Two different CHD8-knockout clones display similar transcriptome in ESC stage.

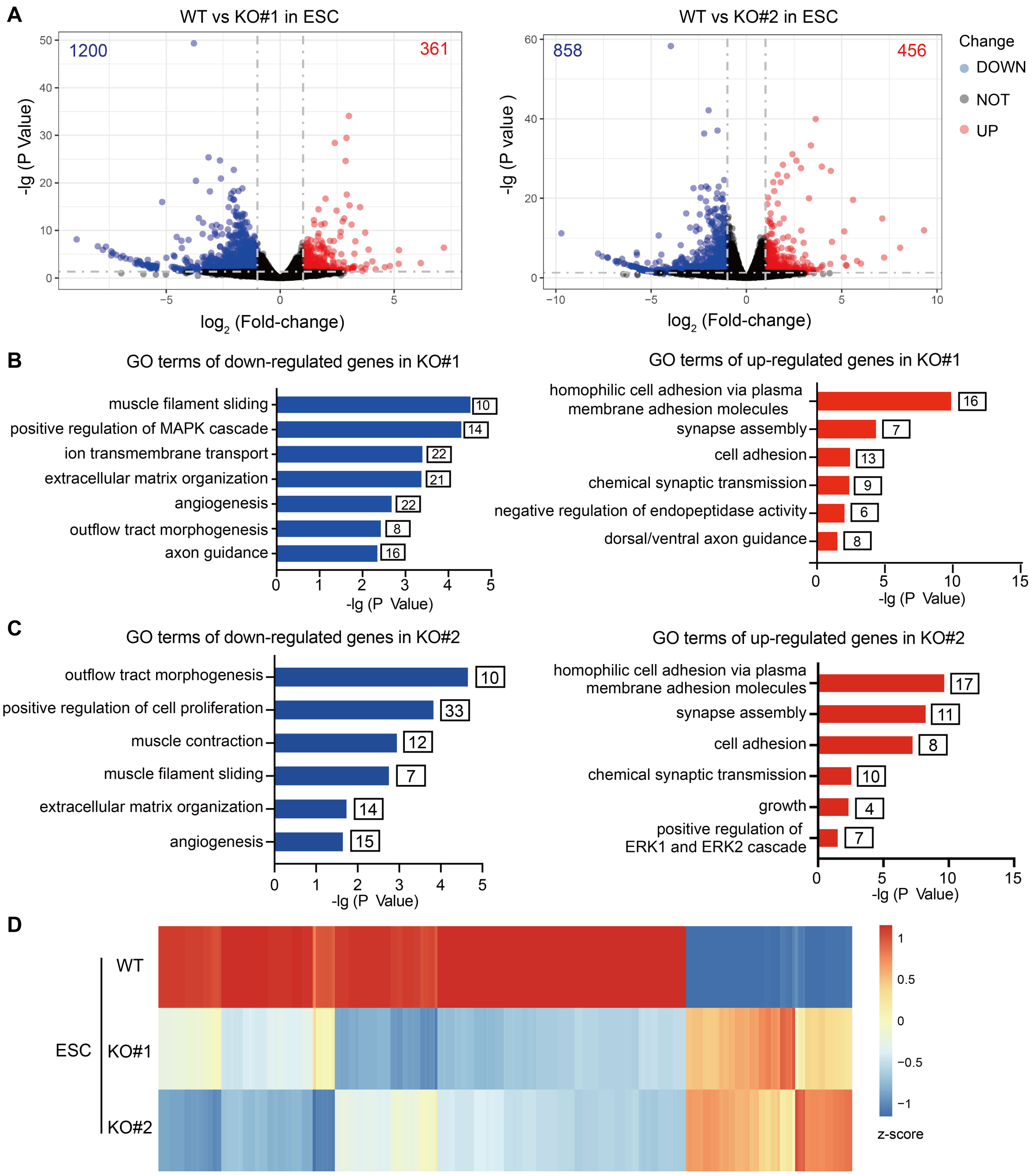

A. Volcano plots show up- and down-regulated genes in CHD8-KO#1 and KO#2 ESCs compared to WT ESC. The bule number displayers the down-regulated genes and the red number displays the up-regulated genes. **B-C.** GO terms of down- and up-regulated genes in CHD8-KO#1 and #2 ESCs compared to WT. **D**. The heatmap of the overlap DEGs in KO#1 and KO#2 ESC (156 genes down-regulated and 453 genes up-regulated).

Figure S2. Two different CHD8-knockout clones display similar transcriptome in NPC stage.

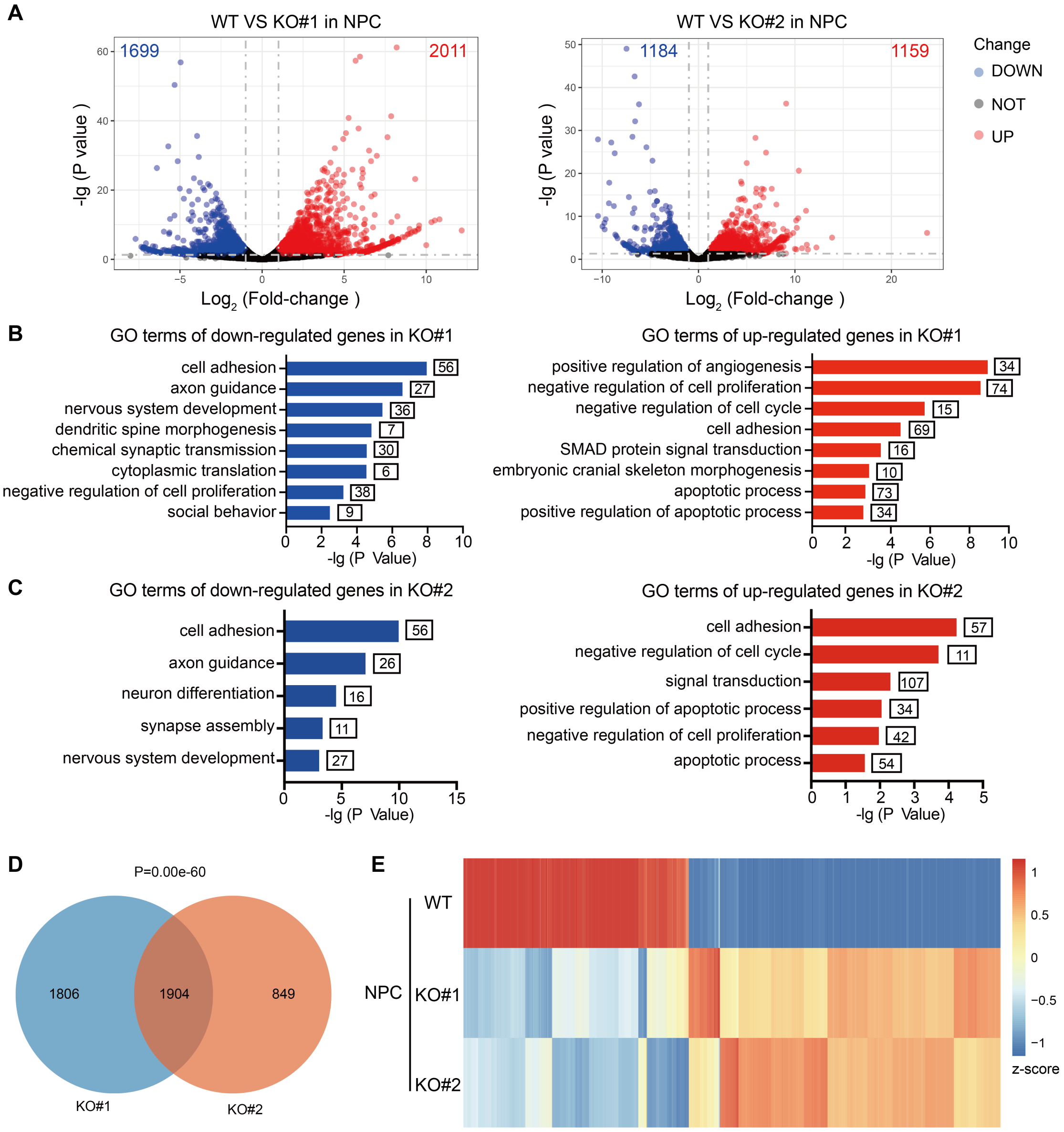

A. Volcano plots show up- and down-regulated genes in CHD8-KO#1 and KO#2 NPCs compared to WT NPCs. The bule number displayers the down-regulated genes and the red number displays the up-regulated genes. **B-C.** GO terms of down- and up-regulated genes in CHD8-KO#1and KO#2 NPCs. **D.** Venn diagram showing the number and P value of overlap DEGs between CHD-KO#1 and KO#2 compared to WT. **E.** The heatmap of the overlap DEGs in KO#1 and #2 NPCs (800 down-regulated genes and 1106 up-regulated genes).

Figure S3. Comparison of expression patterns between CHD8-KO and other CHD8 haploinsufficient models and the ASD-risk gene sets.

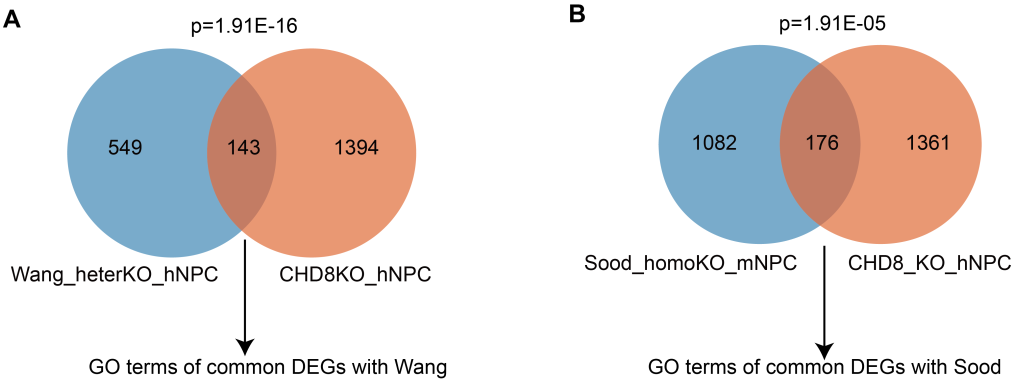

GO terms of common DEGs with Wang

GO term	P value	Example genes
cell adhesion	7.84E-10	*SRPX, COL16A1, LAMA2, TNFAIP6, LAMC3, COL12A1*
extracellular matrix organization	6.31E-09	*COL16A1, LAMA2, LAMC3, LAMC2, COL5A1, COL4A6*
axon guidance	3.00E-04	*EFNA1, CXCL12, LAMA2, UNC5A, BDNF, LHX2, OTX2*
positive regulation of cell migration	7.21E-04	*PDGFRB, TNFAIP6, PODXL, LEF1, PRKCA, LAMC2, ROR2*
forebrain neuron differentiation	3.96E-02	*LEF1, LHX5*

GO terms of common DEGs with Sood

GO term	P value	Example genes
cell adhesion	1.04E-05	*LAMB3, ITGA2, LAMC2, THBS2, MSLN, GRHL2*
positive regulation of cell migration	4.37E-04	*SEMA5B, WNT5B, FAM110C, SEMA6D, ONECUT1, LAMC2*
negative regulation of cell proliferation	5.36E-03	*DLL4, SFRP1, ADGRG1, ZEB1, TES, WARS*
neuron differentiation	1.37E-02	*PTPRD, RTN1, WNT5B, WNT5A, SOX11*
cell differentiation	1.48E-02	*ILDR2, NANOG, FGF1, DLL1, MYT1, DLK1*

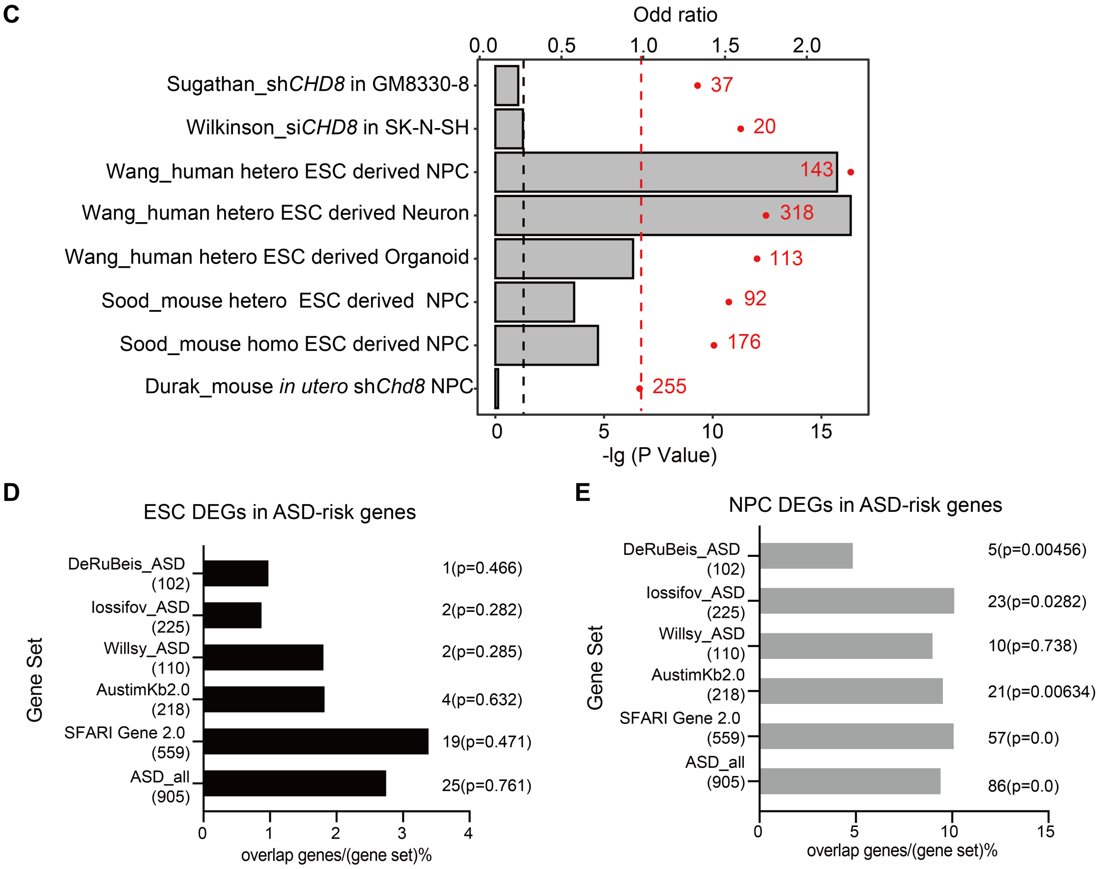

A-B. Top, Venn diagram showing the number and P value of overlap DEGs between Wang and Sood NPC data with our CHD8-KO NPC data; Bottom, GO analysis of the overlap DEGs. **C.** The fisher exact test (one tailed) between our CHD8-KO DEG in NPCs and other CHD8 models. **D-E.** GSEA result of our data in the five ASD-risk gene sets with ESC and NPC DEGs. The number below the gene set represents the size of gene set, and the number next to dot shows the overlap between the identified DEGs and the ASD gene sets.
