# Supplementary figures and images for: CHD8 safeguards early neuroectoderm differentiation in human ESCs and protects from apoptosis during neurogenesis
Source: Cell Death Dis. 2021 Oct 22;12(11):981. doi: 10.1038/s41419-021-04292-5 (PMC8536677; doi:10.1038/s41419-021-04292-5)

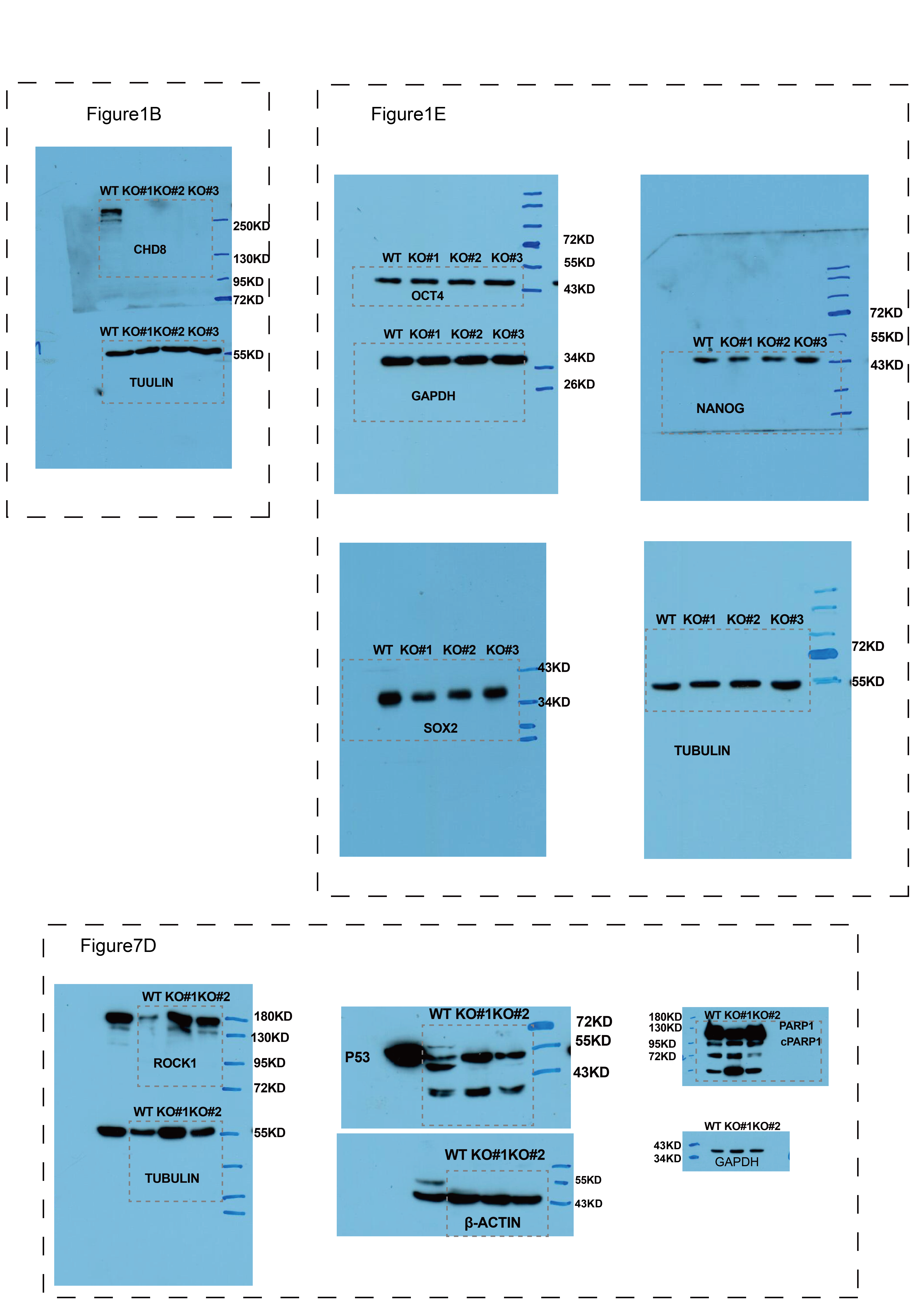

Supplement: Supplementary file 2 — Original data western blot [file 41419_2021_4292_MOESM2_ESM.png]
